# Nurses’ clinical competency and its correlates: before and during the COVID-19 outbreak

**DOI:** 10.1186/s12912-023-01330-9

**Published:** 2023-05-07

**Authors:** Tian Hui, Mohammad Ali Zakeri, Yaser Soltanmoradi, Neda Rahimi, Sayed Mortaza Hossini Rafsanjanipoor, Majid Nouroozi, Mahlagha Dehghan

**Affiliations:** 1 Affiliated Hospital of Jiangnan University, Wuxi, 214122 China; 2 Social Determinants of Health Research Center, Rafsanjan University of Medical Sciences, Rafsanjan, Iran; 3 Non-Communicable Diseases Research Center, Rafsanjan University of Medical Sciences, Rafsanjan, Iran; 4 Faculty Member, School of Paramedicine, Department of Operating Room Technology, Rafsanjan University Medical of Sciences, Rafsanjan, Iran; 5 Department of Surgical Nursing, Nursing and Midwifery School, Rafsanjan University of Medical, Rafsanjan, Iran; 6 Clinical Research Development Unit, Ali-Ibn Abi-Talib Hospital, Rafsanjan University of Medical Sciences, Rafsanjan, Iran; 7 Nursing Research Centre, Kerman University of Medical Sciences, Kerman, Iran; 8 Department of Critical Care Nursing, Razi Faculty of Nursing and Midwifery, Kerman University of Medical Sciences, Haft-Bagh Highway, Kerman, Iran

**Keywords:** Study, Nursing, COVID-19, Professional competence

## Abstract

**Background:**

Clinical competency is the ability to integrate knowledge, skills, attitudes and values into a clinical situation and it is very important in nursing education, clinical settings, nursing management, and crises. This study aimed to investigate nurses’ professional competence and its correlates before and during the COVID-19 pandemic.

**Methods:**

We conducted this cross-sectional study before and during the COVID-19 outbreak and recruited all nurses working in hospitals affiliated to Rafsanjan University of Medical Sciences, southern Iran, so we included 260 and 246 nurses in the study before and during the COVID-19 epidemic, respectively. Competency Inventory for Registered Nurses (CIRN) was used to collect data. After inputting the data into SPSS24, we analysed them using descriptive statistics, chi-square and multivariate logistic tests. A significant level of 0.05 was considered.

**Results:**

The mean clinical competency scores of nurses were 156.97 ± 31.40 and 161.97 ± 31.36 before and during the COVID-19 epidemic, respectively. The total clinical competency score before the COVID-19 epidemic was not significantly different from that during the COVID-19 epidemic. Interpersonal relationships (*p* = 0.03) and desire for research/critical thinking (*p* = 0.01) were significantly lower before the COVID-19 outbreak than during the COVID-19 outbreak. Only shift type had an association with clinical competency before the COVID-19 outbreak, while work experience had an association with clinical competency during the COVID-19 epidemic.

**Conclusion:**

The clinical competency among nurses was moderate before and during the COVID-19 epidemic. Paying attention to the clinical competence of nurses can improve the care conditions of patients, and nursing managers should improve the clinical competence of nurses in different situations and crises. Therefore, we suggest further studies identifying factors improving the professional competency among nurses.

## Introduction

The COVID-19 disease appeared in Wuhan, China in December 2019 and spread rapidly to other Chinese regions and countries [1]; This disease caused many challenges in various people, both patients and other people, and caused a wave of panic among health workers [2–4]. Iran is one of the first ten countries contaminated by this disease [5]. These conditions highlighted the need for proper response of medical staff and hospitals to incidents caused by crises [6, 7]. On the other hand, attention to risk assessment and analysis of the type of health care should be considered [8–10].

The high prevalence of SARS-Cov-2 indicates the importance of clinical competency among health professionals [11]. Studies suggest that nurses are unready, shocked, and confused in emergency situations [12]; therefore, it is important to increase their clinical competency. Clinical competence is one of the important components of nursing care, which has received more attention from health managers [13–15]. Nurses’ clinical competency is a significant issue in various medical fields, with several factors having roles in paying attention to clinical competence among nurses, including rapid changes in healthcare systems, the need to provide safe and cost-effective services, improvement of the level of community health awareness, expectations for receiving higher quality services, and the desire of health organizations to use competent health workforce. Clinical competency includes moral and value dimensions and represents science and skill; honesty, accuracy, communication skills, and adaptability are the main indicators of professional competence [16, 17].

Clinical competence is to use technical and communication skills, knowledge, clinical reasoning, emotions and values ​​in clinical settings. It also refers to the ability to carry out professional functions effectively in the area of practice [18–20]. The World Health Organization (WHO) refers to providing quality health services at different levels [21], and clinical competence has been an important factor in patients’ surgical results, safety, and satisfaction [22]. According to research, an increase in clinical competence increases patient satisfaction [23], and it has a relationship with critical thinking and the level of organizational commitment [24, 25]. Individual and organizational factors affecting nurses’ competences include knowledge and skill, observance of professional ethics, respectful interaction with colleagues, work experience, appropriate communication, interest in the profession and responsibility, educational and clinical setting, and an efficient educational system [26]. Najafi et al. (2022) considered work experience, age, clinical experience in the current ward, higher education level, work while studying, and emotional intelligence as the personal factors affecting nurses’ clinical competences. They found that the environmental-organizational factors, identification of patients' culture and provision of care based on their culture, job satisfaction and consultation with colleagues were effective on nurses’ clinical competences [19]. However, clinical competence may change in emergency situations and crises. The impact of COVID-19 on educational processes, curriculum, and medical education programs has been identified, which can affect competency in care [27].

The COVID-19 outbreak led to a public health emergency of international concern and mainly affected healthcare workers, particularly nurses. Studies reported many mental disorders in healthcare workers and nurses working in high-risk settings, such as anxiety, social problems [28], posttraumatic stress [29], anger, mental health problems [30, 31] and burnout [32], which in turn affected clinical practice and competence among nurses. The COVID-19 pandemic challenged frontline nurses’ personal and professional lives; they were at risk of the COVID-19 disease due to daily nursing care and direct contact with patients, underwent heavy workload, and faced problems in their daily lives [33, 34]. A study in China indicated that nurses perceived the knowledge of COVID-19 well, but most of them lacked work experience in isolation and coronavirus wards, which in turn affected their clinical competences [35]. Since nursing competence plays an important role in the quality of nursing services, particularly in crises, it is crucial to evaluate clinical competency and its correlates during the COVID-19 pandemic. Healthcare systems can use such evaluations to increase their awareness because these evaluations present useful information to address gaps in knowledge and skills and help nurses provide better and more comprehensive care during pandemics. Therefore, the present study aimed to investigate nurses’ professional competences and their correlates before and during the COVID-19 pandemic in order to gain a better insight into the factors affecting the professional competencies of nurses in crises in order to increase it.

## Method

### Study design and setting

This cross-sectional study investigated nurses’ clinical competences before and during the COVID-19 outbreak in public hospitals (Ali-Ibn Abi-Talib and Moradi) in Rafsanjani, southern Iran.

### Sample size and sampling

Sampling was performed before (from February to May 2019) and during the COVID-19 outbreak (from February to May 2021) by census method. The study population consisted of 435 nurses before the COVID-19 outbreak and 510 frontline nurses during the outbreak in Ali-Ibn Abi-Talib hospitals in Rafsanjan. Nurses in charge of direct care of patients, nurses with one year of experience, and nurses who had clinical experience for at least three months met the inclusion criteria. The exclusion criteria were a history of mental disorders in nurses and an incomplete questionnaire.

Three hundred seven nurses completed the questionnaires before the COVID-19 outbreak, but 260 questionnaires were included in the study after removal of the high missing value (47 questionnaires). Therefore, the effective response rate of frontline nurses before the COVID-19 outbreak was 59.77%. Two hundred and eighty-four nurses completed questionnaires during the COVID-19 outbreak, of which thirty-eight questionnaires were removed due to high missing value. The effective response rate of frontline nurses during the COVID-19 outbreak was 48.23% (*n* = 246); the data of 506 nurses were used in the final analysis. After obtaining the necessary permits, one of the researchers interviewed nurses at their workplaces to complete clinical competency questionnaire.

### Measurement

#### Socio-demographics

Socio-demographic information questionnaire included gender, age, marital status, education level, type of employment, income, work experience, shift type, ward type in department, amount of overtime, and history of illness.

#### Competency Inventory for Registered Nurses (CIRN)

The 55-item CIRN was developed and used by Liu et al. in China (Macau, China) to assess the nurses’ clinical competence in different clinical settings. The inventory includes 7 dimensions: a) clinical care (10 items: 2, 3, 5, 9, 12, 15, 20, 24, 27 and 38), b) leadership (9 items: 13, 14, 28, 32, 33, 36, 39, 43 and 48), c) interpersonal relationships (8 items: 4, 18, 22, 23, 30, 34, 35 and 54), d) ethical/legal performance (8 items: 10, 11, 25, 31, 37, 44, 45 and 49), e) professional development (6 items: 6, 26, 29, 52, 53, and 55), f) coaching/training (6 items: 8, 17, 19, 40, 41 and 46), and g) desire for research / critical thinking (8 items: 1, 7, 16, 21, 42, 47, 50 and 51). The CIRN was scored on a five-point Likert scale ranging from 0 to 4 (0 = lack of competence, 1 = low competency, 2 = limited competency, 3 = sufficient competence, and 4 = very high competence), with a higher score indicating a higher competency (high competency: 165–220, moderate competency: 110–165, and low qualification: less than 110). The total score of this questionnaire varies from 0 to 220.

Liu et al. reported Cronbach's alpha coefficient of 0.908 (ranging from 0.718 to 0.903) for the internal consistency of CIRN questionnaire [36]. Ghasemi et al. (2014) in Iran translated this questionnaire into Persian and confirmed its validity and reliability by Cronbach's alpha coefficient of 0.87 for the whole CIRN questionnaire (0.88–0.97 for subscales) [37]. According to Zakeri et al. (2021), Cronbach's alpha coefficients for clinical care, leadership, interpersonal relationships, ethical/legal performance, professional development, coaching/training, desire for research/critical thinking and the overall scale were 0.88, 0.86, 0.85, 0.82, 0.84, 0.83, 0.84 and 0.97, respectively [38]. In the present study, Cronbach's alpha coefficients for the CIRN questionnaire was 0.94.

### Data collection

After obtaining the necessary permits, the researcher went to the Ali-Ibn Abi-Talib Hospital in Rafsanjan city and started sampling. All eligible people were invited to participate in the study and they were asked to complete the questionnaire when they were ready. The data collection process was done during office hours and when the nurses had enough time to complete the questionnaire. The participant could answer the questions with the interviewer. Ali-Ibn Abi-Talib Hospital was the only hospital in Rafsanjan city dedicated to the care of COVID-19 patients.

### Statistical analysis

We input the data into SPSS 24 to analyze them. Frequency, percentage, mean and standard deviation were used to define the dimensions of clinical competence and demographic characteristics. Independent t test was used to comparison of the clinical competency ant its dimensions before and during the COVID-19 among nurses. Multivariate logistic regression was used to investigate the relationship between the variables of analysis and clinical competence of nurses. Significance level was considered to be 0.05.

## Results

The samples before the COVID-19 outbreak included 260 nurses with a mean age of 32.98 ± 6.13 years. Most of them were female (*n* = 214, 82.3%), married (*n* = 221, 85.0%), employed (*n* = 157, 60.3%), had a bachelor's degree in nursing (*n* = 235, 90.4%), rotating shifts (236, 90.8%), and 5–10 years of work experience (122, 46.9%). The samples during the COVID-19 outbreak included 246 nurses with a mean age of 35.85 ± 7.68 years. Most of them were female (*n* = 166, 67.5%), married (*n* = 183, 74.4%), employed (*n* = 134, 54.5%), had a bachelor's degree in nursing (*n* = 211, 85.8%), rotating shifts (203, 82.5%), and 31–60 h of overtime per month (95, 38.7%) (Table 1).

**Table 1:** The relationship between participants’ demographic characteristics and clinical competency (before and during the COVID-19)

Group	Before COVID-19 (*n* = 260)			During COVID-19 (*n* = 246)		
Variables	Frequency (Valid percent)	Clinical competency		Frequency (Valid percent)	Clinical competency	
		Mean (SD)	Statistical test/*P* value		Mean (SD)	Statistical test/*P* value
**Gender**						
Male	46 (17.7)	155.28 (28.89)	t = -0.40 (0.68)	80 (32.5)	158.00 (30.09)	t = -1.37 (0.17)
Female	214 (82.3)	157.34 (31.97)		166 (67.5)	163.88 (32.15)	
**Age (yr.)**						
≤ 30	99 (38.1)	154.22 (33.60)	t = -1.11 (0.26)	73 (29.7)	156.08 (26.98)	t = -2.07 (0.04)
> 30	161 (61.9)	158.67 (29.95)		173 (70.3)	164.45 (33.06)	
**Marital status**						
Married	221 (85.0)	156.43 (34.68)	t = -0.11 (0.90)	183 (74.4)	155.85 (28.43)	t = -1.79 (0.07)
Unmarried/other	39 (15.0)	157.07 (30.87)		63 (25.6)	164.07 (32.37)	
**Education level**						
Bachelor	235 (90.4)	157.39 (31.23)	t = 0.65 (0.51)	211 (85.8)	161.32 (31.72)	t = -0.79 (0.42)
Masters	25 (9.6)	153.08 (33.40)		35 (14.2)	165.88 (30.73)	
**Type of employment**						
Hired	157 (60.3)	157.68 (30.38)		134 (54.5)	163.38 (33.39)	F = 0.32 (0.73)
Contract recruiters^a^	68 (26.2)	159.07 (33.09)	H = 1.87 (0.39)	87 (35.4)	160.08 (30.20)	
Committed^b^	35 (13.5)	149.71 (32.46)		25 (10.2)	160.96 (26.34)	
**Income (Million Toman)**						
< 4	105 (40.4)	153.93 (34.75)	F = 1.10 (0.33)	16 (6.5)	171.37 (23.43)	F = 0.78 (0.46)
4 – 7	136 (52.3)	158.33 (28.27)		77 (31.3)	160.70 (31.97)	
> 7	19 (7.3)	164.05 (33.15)		153 (62.2)	161.62 (32.09)	
**Work experience (yr.)**						
< 5	75 (28.8)	156.17 (33.93)	F = 2.11 (0.09)	65 (26.4)	161.76 (27.00)	F = 3.32 (0.02)
5 -10	122 (46.9)	153.22 (30.05)		63 (25.6)	153.92 (30.15)	
11 – 15	35 (13.5)	163.74 (29.39)		63 (25.6)	161.46 (35.16)	
> 15	28 (10.8)	167.00 (30.68)		55 (22.4)	172.01 (31.83)	
**Shift type**						
Fixed	24 (9.2)	168.50 (30.53)	t = 1.89 (0.05)	43 (17.5)	160.79 (35.35)	t = -0.27 (0.78)
Rotating	236 (90.8)	155.80 (31.31)		203 (82.5)	162.22 (30.78)	
**Ward**						
Critical/intensive	73 (28.1)	158.35 (30.23)	H = 2.18 (0.33)	56 (22.8)	165.32 (29.64)	H = 0.92 (0.63)
Emergency	146 (56.1)	158.13 (32.22)		155 (63.0)	161.66 (31.92)	
Medical	41 (15.8)	150.41 (30.40)		35 (14.2)	157.97 (33.23)	
**Overtime (h)**						
0 – 30	24 (9.2)	162.83 (29.82)	F = 1.21 (0.30)	33 (13.4)	156.78 (28.09)	F = 1.09 (0.35)
31 – 60	59 (22.7)	158.20 (31.38)		95 (38.7)	163.62 (32.34)	
61 – 90	142 (54.6)	157.60 (31.96)		81 (32.9)	159.22 (32.13)	
> 90	35 (13.5)	148.34 (29.82)		37 (15.0)	168.37 (31.01)	
**Illness**						
Yes	41 (15.8)	162.46 (32.97)	t = 1.22 (0.22)	48 (19.5)	159.75 (32.67)	t = -0.54 (0.58)
No	73 (84.2)	155.94 (31.07)		198 (80.5)	162.51 (31.34)	

*SD* Standard Deviation, *t* Independent t test, *F* Analysis of variance, *H* Kruskal–Wallis H, *a* Annually contracted with payment similar to hired nurses, *b* It is obligatory to work for government for two years at a lower rate of pay

**Fig. 1:**
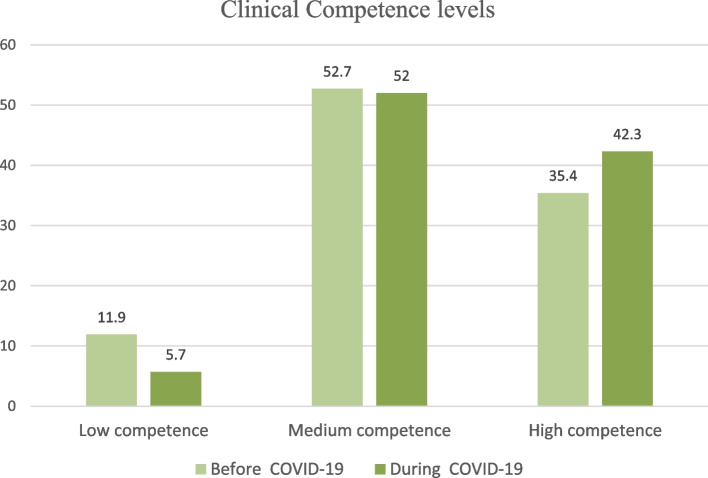
The comparison of the levels of clinical competency before and during the COVID-19

The mean scores of nurses’ clinical competency before and during the COVID-19 were 156.97 ± 31.40 and 161.97 ± 31.56, respectively, with clinical care and professional development subscales receiving the highest and lowest scores. The interpersonal relationships (*p* = 0.03) and desire for research/critical thinking (*p* = 0.01) scores were significantly lower before the COVID-19 outbreak than during the COVID-19 outbreak. The nurses' total clinical competency scores did not change significantly before and during the COVID-19 outbreak (*p* > 0.05) (Table 2). Before the COVID-19 outbreak, 11.9% had low, 52.7% had moderate, and 35.4% had high level of clinical competency, while during the COVID-19 outbreak, 5.7% had low, 52% had moderate, and 42.3% had high level of clinical competency (χ^2^ = 5.66, *p* = 0.01) (Fig. 1).

**Table 2:** Comparison of the clinical competency ant its dimensions before (*n* = 260) and during the COVID-19 (*n* = 246)

Group	Before COVID-19			During COVID-19			Independent t test	Effect size	*P* value
**Variables**	**Median**	**Mean**	**SD**	**Median**	**Mean**	**SD**			
Clinical care	29.0	28.39	6.28	30.0	29.39	6.12	-1.82	0.16	0.06
Leadership	27.0	26.51	5.00	27.0	27.15	5.34	-1.37	0.12	0.17
Interpersonal relationships	23.0	23.03	5.02	24.0	23.94	4.68	-2.11	0.18	**0.03**
Ethical/legal performance	23.0	22.90	4.57	24.0	23.69	4.89	-1.87	0.16	0.06
Professional development	17.0	16.74	3.97	17.0	16.86	4.38	-0.31	0.02	0.75
Coaching/training	18.0	17.16	3.69	18.0	17.64	3.74	-1.45	0.12	0.14
Desire for research/critical thinking	23.0	22.21	5.00	23.0	23.28	4.84	-2.42	0.21	**0.01**
Total Clinical Competency	159.0	156.97	31.40	162.0	161.97	31.56	-1.78	0.15	0.07

*SD* Standard Deviation

We found no significant difference in demographic characteristics and clinical competency before the COVID-19 outbreak, while age and work experience had a significant association with clinical competency during the COVID-19 outbreak (*p* = 0.009) (Table 1). We used multiple regression with backward method for further analysis and included all variables with *p*-value < 0.3 in the multivariate regression model. The results indicated a relationship between shift type and clinical competency before the COVID-19 outbreak, as well as between work experience and clinical competency during the COVID-19 outbreak (Table 3).

**Table 3:** Multiple models of the associations between variables and clinical competency before and during the COVID-19 outbreak

**Variable**			**B**	**SE**^a^	**β**	**T**	***P***	**95% CI** **Lower**	**95% CI** **Upper**	**R**^**2**^
**Clinical competency**	**Before COVID-19**	Constant	191.95	14.19	-	13.52	< 0.001	163.99	219.90	% 2
		Shift type	-13.37	6.67	-0.12	-2.00	0.046	-26.52	-0.22	
		Overtime	-0.133	0.07	-0.11	-1.79	0.074	-0.27	0.01	
	**During COVID-19**	Constant	151.01	11.08	-	13.62	< 0.001	129.18	172.85	% 2.6
		Work experience	5.14	1.93	0.181	2.65	0.009	1.32	8.96	
		Gender	7.88	4.30	0.117	1.83	0.068	-0.58	16.36	
		Income	-5.78	3.47	-0.113	-1.66	0.097	-12.62	1.05	

^a^Standard error; CI, Confidence intervals for B; Work experience (1 =  > 5, 2 = 5 -10, 3 = 11 – 15, 4 =  > 15); Shift type (1 = Fixed and 2 = Not fixed)

## Discussion

The present study aimed to investigate nurses’ professional competency and its correlates before and during the COVID-19 pandemic. The study results indicated no significant difference in the total scores of nurses’ clinical competence before and during the COVID-19 epidemic and nurses received moderate clinical competence in both situations. Ahmadi et al. (2022) supported our results and reported nurses’ moderate clinical competence in the COVID-19 wards [21], but Alan et al. (2022) in Turkey found that nurses’ professional competences in the COVID-19 wards were above the average level [39]. Different results may be due to the different research settings, hospital conditions, nurses’ working conditions in the wards, and managers’ supports from nurses in different dimensions during the epidemic.

Faraji et al. (2019) [40] and Jalali et al. (2019) [41], as well as Kalantary et al. (2016) [42] reported a high level of clinical competence among Iranian nurses working in intensive care units. Kajander-Unkuri et al. (2014) revealed good level of the clinical competence in newly graduated nursing students [43], but these studies were not consistent with the present study. Nurses’ low clinical competence in our study may be due to their problems during the COVID-19 epidemic and their lack of time to acquire professional skills to care for these patients [44].

Low clinical competence among nurses working in the COVID-19 wards can be due to stressful working conditions, high patient mortality rates, and the need to have high skills. Labrague et al. (2021) reported an association between fears of COVID-19, decreased job satisfaction, and increased psychological distress. They mentioned a high level of fear of COVID-19 among nurses, who were not full-time and did not attend COVID-19 training courses. Labrague argued that nurses working in COVID-19 wards were at a higher risk of infection than the general population, so they were more afraid of transmitting the disease to their family members and friends. An increase in the number of admitted patients, social distancing, and quarantine might exacerbate this condition and affected clinical skills [45].

An important point in our study was that we found no difference in nurses’ clinical competences before and after the COVID-19 epidemic and their clinical competences did not decrease during the COVID-19 outbreak. This result suggests that nurses do their best to take care of patients even in critical conditions. Jang and Cho (2022) reported disaster nursing competencies correlated with age, nursing career, compassion satisfaction, and secondary traumatic stress [46]. Arshadi Bostanabad et al. (2022) reported clinical competency has been tied to nurse health and quality of care [47]. The review of the literature showed that although some studies have mentioned the clinical qualifications of nurses in the COVID-19 epidemic, no comparison has been made with before the crisis of the COVID-19, so there is a need for further investigation in this regard.

Our results indicated that the subscales of clinical care and professional development received the highest and lowest scores, respectively. The scores of interpersonal relationships and desire for research/critical thinking before the COVID-19 outbreak were significantly lower than that during the COVID-19 outbreak. Ahmadi et al. (2022) and Saadati et al. (2018) supported our results [21, 48]. Therefore, the ability to become empowered in clinical care was very important from nurses’ perspectives, but their low scores of professional developments indicate that they must become empowered in other dimensions, particularly during the epidemics and crises because they spend most of their time taking care of patients and ignore other areas.

However, Fotohi et al. (2019) did not confirm our results because personal management and practical competence, and desire for research received the highest and lowest scores in their study [49]. Kalantary et al. (2016) did not support our study and reported that the quality assurance and occupational and organizational duties had the lowest and highest scores, respectively [42]. They did not conduct their study during the COVID-19 pandemic that might have a positive effect on nurses’ practices, so the nurses participating in the present study focused on clinical care more. Another reason for different results is that nurses may set different priorities based on their positions, type of hospital, type of management governing their workplaces and the wards where they are working.

The study results showed an association between shift type and clinical competence before the COVID-19 outbreak, as well as between work experience and clinical competence during the COVID-19 outbreak. Keshavarzi et al. (2021) demonstrated that the type of shift work had a significant relationship with nurses’ overall clinical skills in neonatal intensive care units [50] (36). Arshadi Bostanabad et al. (2022) found a positive and significant relationship between the clinical competence and work experience of the nurses working in the intensive care unit who cared for patients with COVID-19 [51]. Faraji et al. (2019) also indicated a significant relationship between work experience and clinical competence [40]. Manoochehri et al. (2015) studied the clinical competence among nurses working in the hospitals affiliated to Hormozgan University of Medical Sciences in southern Iran and reported that experienced nurses were more clinically competent than novice nurses [52]. Liou et al. (2013) revealed that work experience increased clinical competence [53]; Istomina et al. (2011) believed that nurses’ experience and training led to their professional developments, strengthened their learning, and increased their skills [54]. Blomberg et al. (2019) demonstrated that work experience insured development of clinical competency [55]. When allocating patients with sensitive conditions in different wards, including COVID-19, nurse managers must remember that nurses with more work experiences will be more clinically competent.

However, Bahreini et al. (2011) reported no significant relationship between work experience and clinical competence [56]. As studies on the COVID-19 are limited, further studies are necessary to determine the correlation between work experience and clinical competence during the COVID-19 outbreak. Qualitative studies with the aim of discovering the roots of unexpected results can be useful.

### Limitations

This study had some limitations: we studied nurses living in a city in southeastern Iran, so the generalization of the results to other societies should be done with caution due to the cultural and social differences. Another limitation was the economic, social and cultural conditions of the participants, which were beyond our control. In the review of the literature, it was found that no study was found to examine the clinical qualifications of nurses during and before the COVID-19 outbreak, so caution should be taken in interpreting the results.

## Conclusion

According to the study results, work experience can be one of the factors influencing the clinical competence among nurses. Nursing as a clinical discipline is developing and nurses are key members in various settings. Nurses must maintain their professional competences and evaluate and prioritize their clinical competence indicators to improve the healthcare system. Evaluation of clinical competence is particularly important in critical situations, which can improve patients’ condition. Therefore, we suggest policymakers and nurse managers recognizing and increasing nurses’ clinical competences, particularly in critical situations so that they can provide more correct and effective care to the patients. Future studies should focus on the recognition of factors and critical conditions affecting the clinical competence of nurses.

## Data Availability

The datasets used and/or analysed during the current study available from the corresponding author on reasonable request.
